# Soil Microarthropod Communities Along Salt Marsh Transects of the Wadden Sea Are Predominantly Structured by Niche Differentiation

**DOI:** 10.1002/ece3.73245

**Published:** 2026-03-20

**Authors:** Xue Pan, Lara Warnke, Lennart Zwolski, Maria Rinke, Ting‐Wen Chen, Stefan Scheu, Mark Maraun

**Affiliations:** ^1^ JFB Institute of Zoology and Anthropology University of Göttingen Göttingen Germany; ^2^ Centre of Biodiversity and Sustainable Land Use University of Göttingen Göttingen Germany

**Keywords:** Collembola, community assembly, deterministic vs. stochastic processes, heterogeneity, Mesostigmata, niche, Oribatida

## Abstract

The relative importance of deterministic versus stochastic processes in community assembly is widely discussed in ecology. Stochastic models explain community assembly by ignoring the niches of species, whereas deterministic models assume the niche concept to be key for understanding the assembly of species. Whether stochastic or deterministic processes dominate in shaping the community composition of soil animals is gaining increased attention. Here, we investigated the spatial heterogeneity of soil microarthropod communities (Collembola, Oribatida, Mesostigmata) along a salt marsh transect from the upper salt marsh (USM) to the lower salt marsh (LSM) to the pioneer zone (PZ) on three islands, that is, Norderney, Spiekeroog, and Wangerooge, in the Wadden Sea of Germany. We hypothesized that microarthropod communities are predominantly structured by niche‐based processes and change in a deterministic way from the USM to the LSM to the PZ, whereas microarthropod communities differ little between the three islands of similar history and position in the Wadden Sea. Supporting our hypothesis, Oribatida and Mesostigmata communities differed more strongly between the three zones than between the islands indicating that environmental factors changing along the saltmarsh gradient select for specific mite communities in a deterministic way. Collembola communities also differed between the three zones but also between the islands indicating that they are structured by both environmental filtering as well as stochasticity. However, even the differences in mite and Collembola communities between the islands may—at least in part—be explained by environmental filtering, for example, by differences in nutrient input from the Ems river estuarine. Overall, the results indicate that stochasticity plays only a limited role in structuring microarthropod communities in the dynamic marine—terrestrial boundary of salt marshes pointing to the importance of niche differentiation and environmental filtering.

## Graphical abstract

We combined α‐ and β‐diversity to investigate the community assembly of soil microarthropods (Collembola, Oribatida, Mesostigmata) along a salt marsh transect from the upper salt marsh to the lower salt marsh to the pioneer zone on three islands in the Wadden Sea of Germany. The results suggest that soil microarthropod communities in the dynamic marine–terrestrial boundary of salt marshes are predominantly structured by deterministic factors.

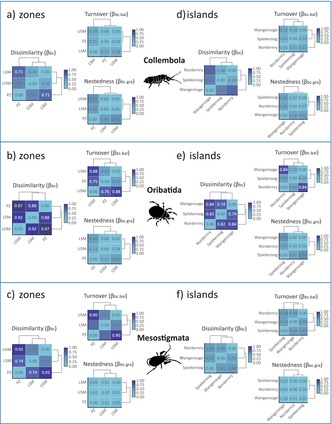

## 1 Introduction

Biodiversity is shaped by random processes such as stochastic colonization and extinction events, and by deterministic processes due to environmental filtering based on the niche of species (MacArthur and Wilson 1967; Hutchinson 1957). In contrast to stochastic models, deterministic theories propose increased environmental heterogeneity to positively affect biodiversity due to the availability of a wider range of niches (Hutchinson 1957; Shmida and Wilson 1985; Chase and Leibold 2003; Loke and Chisholm 2022). In addition to fostering coexistence of species due to the provisioning of a wider range of niches (Tews et al. 2004; Stein et al. 2014), heterogeneous environments may favor the persistence of species due to the availability of shelter and refuges (Kallimanis et al. 2010; Stein et al. 2014), and the increase in the probability of speciation due to isolation and adaptation (Rosenzweig 1995; Hughes and Eastwood 2006; Stein et al. 2014). Deterministic theories propose that communities inhabiting similar systems (offering similar niches) are predictable due to species occupying the same niches.

Generally, the relative importance of stochastic and deterministic factors driving community assembly is widely discussed in ecology (Hubbell 2001; Farnon Ellwood et al. 2009; Chen et al. 2014). However, it has been little studied in soil animals. Soils are diverse systems in which driving factors affecting biodiversity and community assembly processes are still little investigated. Recently, it has been emphasized that stochastic processes may be more important in explaining soil animal community composition than previously assumed (Caruso et al. 2012; Lindo et al. 2023). However, to allow evaluating their relative importance compared to deterministic processes for soil animal community assembly, further investigations are needed (Ingmarsdottir et al. 2012; Thakur et al. 2020; Lindo et al. 2023). Existing studies generally assume niche‐based approaches to be more powerful in explaining shifts in community composition than stochastic approaches, but this likely varies between ecosystem types (Caruso et al. 2012; Magilton et al. 2019; Noske et al. 2024).

Soil microarthropod communities mainly consist of Collembola (Hexapoda), Oribatida (Acari), and Mesostigmata (Acari), with Oribatida and Collembola species predominantly living as decomposers and Mesostigmata as predators. The former two groups play a pivotal role in soil by contributing to decomposition and nutrient cycling, and the latter represents a dominant group of predators in soil. Each of the groups typically is locally abundant and contributes substantially to local soil animal diversity; however, the mechanisms contributing to their high local diversity are little understood (Nielsen 2019; Van Straalen 2023). Understanding which factors shape their communities is fundamental in explaining distribution patterns and diversity of communities across different systems. Microarthropod species were, for long, thought to be mainly food generalists (Anderson 1977; Rusek 1998; Koehler 1999). In recent years, however, it has been realized that they occupy distinct trophic niches (Chahartaghi et al. 2005; Klarner et al. 2013; Maraun et al. 2023), challenging the assumption that they typically occupy broad ecological niches.

Salt marshes are transitional habitats between the tidal mudflats and coastal dunes along shores of marine waters. Salt marshes display a typical zonation from the upper salt marsh to the lower salt marsh to the pioneer zone and form a natural gradient of increasing frequency of inundation (Haynert et al. 2017). This zonation leads to high environmental heterogeneity, as the frequency of inundation imposes environmental harshness due to pronounced changes in temperature, moisture, and salinity thereby shaping plant and animal communities (Bockelmann et al. 2002; Vöge et al. 2008; Haynert et al. 2017). Salt marsh islands of the Wadden Sea developed from sedimentation processes along the southern coast of the North Sea, which is an ongoing process. Along the Wadden Sea, a chain of islands has been formed spanning from the Netherlands in the west to Denmark in the northeast. The islands are relatively young (about 8000 years old; Reise et al. 2010) and due to their similar formation, size, and location can be compared in a straightforward way. This allows investigating if communities on these islands, in particular in the dynamic salt marsh habitats, are structured by random processes driven by stochasticity or by deterministic factors such as the availability of different niches along salt marsh transects.

We compared microarthropod communities including Collembola, Oribatida, and Mesostigmata along a transect from the upper salt marsh to the lower salt marsh to the pioneer zone spanning major environmental heterogeneity gradients. The study was performed on three islands along the German Wadden Sea coast, that is, Norderney, Spiekeroog, and Wangerooge, which are of similar size and age. Assuming the prevalence of niche‐based assembly processes, we hypothesized that (1) microarthropod communities strongly differ between the three salt marsh zones due to strong changes of environmental factors along the gradient selecting for certain species, and (2) microarthropod communities differ little between the three islands underlining the prevalence of niche‐based filtering of species.

## 2 Material and Methods

### 2.1 Study Site and Sample Collection

Samples were taken on the islands of Norderney (53°41′14.4″–53°43′51.2″ N, 7°08′07.6″–7°20′57.0″ E), Spiekeroog (53°45′2″–53°47′1″ N, 7°40′0″–7°49′1″ E), and Wangerooge (53°45′02.0″–53°47′46.3″ N, 7°40′00.0″–7°58′43.6″ E) belonging to the East Frisian Islands and forming part of the Wadden Sea National Park of Lower Saxony (Germany). Sampling was conducted in the framework of the DynaCom project (https://uol.de/en/icbm/collaborative‐projects/dynacom) in October 2020. The salt marshes of the three islands are located on the leeward side facing the back barrier tidal flats. Samples were taken in each of the three salt marsh zones characterized by different plant communities and increasing frequency of inundation (Rinke et al. 2022; Zielinski et al. 2022). The upper salt marsh (USM) is located > 35 cm above the mean high‐water level (MHWL) and is inundated 35–70 times a year. The lower salt marsh (LSM) is located 35–0 cm above the MHWL and is inundated 150–250 times a year. The pioneer zone (PZ) is located below the MHWL and is inundated twice a day. In each zone, five soil cores of the top 5 cm of the soil (including litter) were taken (diameter 5 cm, core area 19.6 cm^2^). This was done in an identical way on each of the three islands resulting in a total of 45 samples.

### 2.2 Extraction and Species Determination

Samples were transported to the laboratory and animals were extracted by heat (Kempson et al. 1963), with the extraction starting < 24 h after the samples had been taken. Animals were sorted to taxonomic groups (Collembola, Oribatida, Mesostigmata) and determined to species level using the keys of Hopkin (2007) for Collembola, Weigmann (2006) for Oribatida, and Karg (1989, 1993) for Mesostigmata.

### 2.3 Statistical Analyses

All statistical analyses were carried out in R 4.3.1 (R Core Team 2023). Rarefaction analysis was performed using the *iNEXT()* function in the ‘iNEXT’ package (Hsieh et al. 2024) for the three islands and the three zones to evaluate if sample effort was adequate for reliable species richness estimation. Prior to the analyses, animal species which occurred in one sample only were excluded. The analyses were performed for Oribatida, Collembola, and Mesostigmata separately. Differences in density and diversity between the islands and salt marsh zones were inspected by analysis of variance (ANOVA) with the fixed factors “Island” and “Salt marsh zone” using the *aov()* function in the ‘stats’ package. Prior to ANOVAs, data were log‐transformed to improve homogeneity of variance.

Shifts in community composition along the salt marsh transects and across the islands were investigated by abundance‐based pairwise dissimilarity using the *beta.pair.abund()* function in the ‘betapart’ package (Baselga 2013, 2017; Baselga et al. 2023). The overall abundance‐based pairwise dissimilarity (*β*_bc_), measured as Bray–Curtis index, was further partitioned into two additive components: (i) turnover component (= balanced variation in abundance, *β*_bc.bal_), whereby individuals of species in one site are substituted by the same number of individuals of different species in another site, and (ii) nestedness component (= abundance gradients, *β*_bc.gra_), whereby individuals are lost from one site to another, that is, *β*_bc_ = *β*_bc.bal_ + *β*_bc.gra_ (Baselga 2013, 2017). To delineate the degree of dissimilarity between soil microarthropod assemblages, cluster heat maps based on the UPGMA dendrograms of β_bc_, β_bc.bal_, and β_bc.gra_ matrices were produced using the *heatmaply()* function in the ‘heatmaply’ package (Galili et al. 2018). The analyses were performed for all three taxa together, but also separately for Collembola, Oribatida and Mesostigmata.

We performed variance partitioning analysis to investigate how much of the variation is explained by the independent factors “Island” (Norderney, Spiekeroog, Wangerooge) and “Salt marsh zone” (USM, LSM, PZ) using the varpart*()* function in the ‘vegan’ package (Oksanen et al. 2022). Subsequently, we used Redundancy Analysis (RDA) with 999 permutations to test if the effects of the two factors “Island” and “Salt marsh zone” were significant.

Shifts in community composition across the islands and along the salt marsh transects were investigated by Nonmetric Multidimensional Scaling (NMDS) using the *metaMDS()* function in the ‘vegan’ package (Oksanen et al. 2022). This resulted in a dataset with two dimensions for Oribatida, three dimensions for Collembola, and four dimensions for Mesostigmata (*dimcheckMDS()* function in the ‘goeveg’ package; Goral and Schellenberg 2021). The values of the NMDS axes were used as dependent variables in MANOVA (*manova()* function in the ‘stats’ package) with “Island” and “Salt marsh zone” as fixed factors. To depict species overlap between zones and islands, Venn diagrams were used (*ggVennDiagram()* function in the ‘ggVennDiagram’ package; Gao et al. 2021).

## 3 Results

### 3.1 Diversity

**FIGURE 1:**
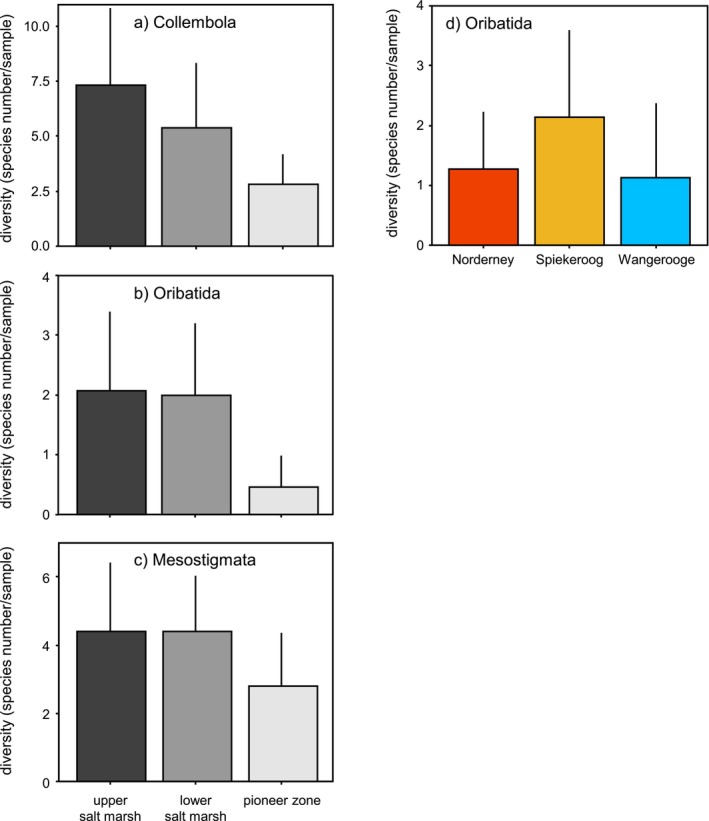
Diversity of (a) Collembola, (b) Oribatida, and (c) Mesostigmata in the upper salt marsh, lower salt marsh, and pioneer zone; and diversity of (d) Oribatida on Norderney, Spiekeroog, and Wangerooge.

In total, 82 soil microarthropod species/taxa were identified (32 Collembola, 13 Oribatida, 37 Mesostigmata; Table S1). Rarefaction analysis indicated that sampling was appropriate for reflecting the number of species on islands and in salt marsh zones (Figure S1). The average number of species of Collembola, Oribatida, and Mesostigmata was lower in the PZ than in the USM and the LSM (ANOVA: *F*_2,31_ = 8.73, *p* < 0.001; *F*_2,36_ = 13.16, *p* < 0.001; *F*_2,36_ = 12.8, *p* = 0.018, respectively; Figure 1a–c). Furthermore, species numbers of Oribatida, but not those of Collembola and Mesostigmata, were higher on Spiekeroog than on Norderney and Wangerooge (ANOVA: *F*_2,36_ = 4.73, *p* = 0.014; Figure 1d, Table S2).

### 3.2 Density

Density of Collembola on Norderney (28,266 ± 28,319 ind./m^2^) and Spiekeroog (27,486 ± 22,119 ind./m^2^) was similar and higher than that on Wangerooge (9671 ± 14,356 ind./m^2^) (ANOVA: *F*_2,31_ = 8.7, *p* < 0.001; Figure S2a). Further, it was higher in the USM (25,111 ± 19,238 ind./m^2^) and LSM (32,067 ± 31,492 ind./m^2^) than the PZ (8653 ± 11,625 ind./m^2^) (ANOVA: *F*_2,31_ = 7.79, *p* = 0.0018; Figure S2b). Density of Oribatida was highest on Spiekeroog (7771 ± 9858 ind./m^2^) where it declined from the USM to the PZ. It was generally lower on Norderney (1527 ± 1875 ind./m^2^) and Wangerooge (1493 ± 2172 ind./m^2^) where it was similar in the USM and the LSM and lower in the PZ (ANOVA: *F*_2,36_ = 3.21, *p* = 0.023 for the “Island” × “Salt marsh zone” interaction; Figure S2c). The density of Mesostigmata neither differed significantly between the three islands (5090 ± 3609, 5022 ± 4859, and 5294 ± 2839 ind./m^2^ for Norderney, Spiekeroog, and Wangerooge, respectively) nor between the salt marsh zones (5497 ± 5003, 5803 ± 3258, and 4106 ± 2710 ind./m^2^ for the USM, LSM and PZ, respectively).

### 3.3 Community Dissimilarity

**FIGURE 2:**
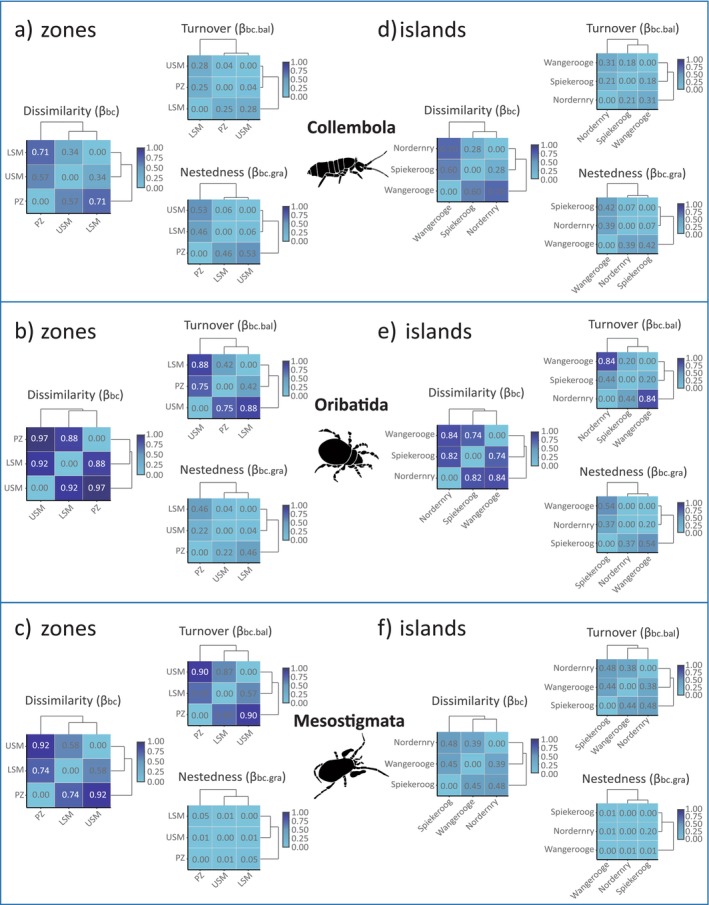
Species‐level dissimilarity matrix heat maps along the salt marsh transect and between the three islands for Collembola (a, d, respectively), Oribatida (b, e), and Mesostigmata (c, f). The Bray–Curtis dissimilarity (*β*_bc_) is partitioned into (i) turnover component (*β*_bc.bal_) and (ii) nestedness component (*β*_bc.gra_). Dendrograms are based on hierarchical cluster analysis (unweighted pair group method with arithmetic averages; UPGMA) of the dissimilarity matrix.

For total microarthropods, the dissimilarity values (*β*_bc_) along the three transect zones as well as between the three islands were high, with turnover (*β*_bc.bal_) contributing more than nestedness (*β*_bc.gra_) (Figure S3). Generally, dissimilarity (*β*_bc_) and turnover (*β*_bc.bal_) along transect zones were higher (*β*_bc_ = 0.63, *β*_bc.bal_ = 0.41; Figure 2a) than between the three islands (*β*_bc_ = 0.54, *β*_bc.bal_ = 0.35).

Along the saltmarsh zones, average dissimilarity values (*β*_bc_) of each of the three microarthropod taxa were high and ranged from 0.92 for Oribatida to 0.75 for Mesostigmata to 0.54 for Collembola (Figure 2a–c). Turnover values (*β*_bc.bal_) accounted for most of the dissimilarity in Oribatida and Mesostigmata, whereas nestedness (*β*_bc.gra_) accounted for most of the dissimilarity in Collembola. Moreover, average dissimilarity values (*β*_bc_) of each taxon along the saltmarsh zones (0.54, 0.92, and 0.74 for Collembola, Oribatida, and Mesostigmata, respectively) were higher than between the three islands (0.53, 0.80, and 0.44 for Collembola, Oribatida, and Mesostigmata, respectively) (Figure 2a–f).

Between the three islands, Collembola and Oribatida had high overall dissimilarity (*β*_bc_) and turnover (*β*_bc.bal_), with averaged *β*_bc_ values of Collembola (0.53) being lower than those of Oribatida (0.80). The high dissimilarity of Oribatida resulted from high turnover (*β*_bc.bal_ = 0.50), whereas the high dissimilarity of Collembola resulted from high nestedness (*β*_bc.gra_ = 0.29) (Figure 2d–f).

### 3.4 Community Composition

Community composition of Collembola and Mesostigmata differed significantly between islands; however, the differences depended on salt marsh zone (significant “Island” × “Salt marsh zone” interaction; MANOVA; Wilks Lambda = 0.36, approx. *F*_4,31_ = 2.95, *p* = 0.002 and Wilks Lambda = 0.46, approx. *F*_4,36_ = 1.79, *p* = 0.041, respectively). On Spiekeroog and Norderney, Collembola community composition in the three salt marsh zones was similar and separated in particular the LSM and PZ on Wangerooge along the first NMDS axis (Figure S4). Mesostigmata communities of each of the three salt marsh zones on the three islands overlapped widely, with the exception of the USM and LSM on Wangerooge, which were separated along the second NMDS axis (Figure S5). Oribatida community composition also differed between islands (MANOVA, Wilks Lambda = 0.45, approx. *F*_2,27_ = 6.21, *p* < 0.001) and salt marsh zones (MANOVA, Wilks Lambda = 0.47, approx. *F*_2,27_ = 5.83, *p* < 0.001) but the interaction was not significant, with the NMDS indicating that the communities were more distinct than those of Collembola and Mesostigmata (Figure S6).

Across all three taxa, 12% of the variance in community composition was explained by “Salt marsh zone” and 10% by “Island” (RDA; *F*_2,42_ = 3.62, *p* = 0.006 and *F*_2,42_ = 3.09, *p* = 0.013, respectively). For the three taxa separately, most variance in community composition was explained by “Salt marsh zone” and less by “Island”, in particular for Mesostigmata (10% vs. 8% in Collembola, 9% vs. 7% in Oribatida, and 16% vs. 1% in Mesostigmata), with the RDA for “Salt marsh zone” (*F*_2,42_ = 3.32, *p* = 0.02, *F*_2,42_ = 5.02, *p* = 0.001, and *F*_2,42_ = 5.02, *p* = 0.001 for Collembola, Oribatida, and Mesostigmata, respectively) being generally more significant than for “Island” (*F*_2,42_ = 2.85, *p* = 0.03, *F*_2,42_ = 2.41, *p* = 0.009, and *F*_2,42_ = 0.96, *p* = 0.477 for Collembola, Oribatida, and Mesostigmata, respectively).

Of the total microarthropod taxa, 16 species (24%) occurred in each of the three zones. Most species exclusively occurred in the USM (19 species, 28%), whereas only 5 (7%) and 3 (4%) exclusively occurred in the LSM and PZ (Figure S7a). By contrast, species overlap between the three islands was higher, that is, 24 species (36%). Only five (7%), nine (13%), and seven (10%) of the species occurred only on Wangerooge, Spiekeroog, and Norderney, respectively (Figure S7b).

## 4 Discussion

We investigated the relative importance of deterministic versus stochastic factors for the assembly of soil microarthropod communities (Collembola, Oribatida, and Mesostigmata) along a transect of increasing inundation frequency from the USM to the LSM to the PZ on three similar islands, Norderney, Spiekeroog, and Wangerooge, in the Wadden Sea of Germany. Adopting a niche‐based perspective, we hypothesized that communities will change more strongly along salt marsh zones than between islands since environmental factors change strongly with changes in inundation frequency but differ little between islands.

### 4.1 Soil Microarthropod Communities Along the Salt Marsh Transect

Microarthropod communities generally differed strongly between the three salt marsh zones (USM, LSM, and PZ) indicating that microarthropod species are adapted to certain environmental conditions and are filtered by local environmental conditions. This supports our first hypothesis that the assembly of soil microarthropod communities predominantly follows deterministic processes based on niche differentiation between species (Caruso et al. 2012; Nielsen 2019).

Parallel to the changes in community structure along the salt marsh transect, density and diversity of each of the three microarthropod taxa declined (although this was not significant for Mesostigmata density). This supports the view that environmental conditions are becoming harsher from the USM to the LSM to the PZ, with the most important factor being the increasing inundation frequency towards the PZ (Haque et al. 2024). However, a number of species of each of the three taxa colonized the PZ documenting that terrestrial soil animals are able to survive the harsh abiotic conditions in the PZ such as frequent inundation and strong wave action, for example, by using plastron‐based respiration and by holding onto substrates with claws or other morphological structures (Krantz and Walter 2009; Pfingstl et al. 2020; Haque et al. 2024). However, essential life functions, such as feeding and reproduction, likely pause during flooding (Schulte 1973; Behan‐Pelletier and Eamer 2007; Rinke et al. 2022, 2023). Moreover, the availability and accessibility of food resources decreases from the USM to the PZ due to the decline in the coverage by terrestrial plants and more frequent flooding frequency. As shown previously, soil arthropods across the studied salt marsh gradient predominantly rely on resources originating from terrestrial plants rather than on resources of marine origin (Rinke et al. 2022, 2023). Furthermore, the high salt concentration across salt marsh zones requires species to generally be halotolerant.

Community dissimilarity along the salt marsh transect was highest in Oribatida, slightly lower in Mesostigmata, and lowest in Collembola, indicating that the taxa respond differently to abiotic environmental gradients. Oribatida in the PZ included several species which are specialized in different ways, for example, in respect to feeding, survival strategies, and reproduction, and most of them only occur in salt marshes. This applies to *Ameronothrus schneideri* , *Hermannia pulchella* , and *Zachvatkinibates quadrivertex* , three species occurring frequently in the PZ and the LSM. Ameronothridae species are uniformly halobionts and characterized by plastron respiration. They tolerate being inundated but resume activity when submerged (Pugh et al. 1990; Bücking et al. 1998; Pfingstl and Krisper 2014), and stable isotope (^15^N, ^13^C) signatures indicate that they feed on marine algae (Haynert et al. 2017; Maraun et al. 2023). Additionally, many species living in intertidal and littoral habitats, including *A. schneideri* , *H. pulchella* , and *Z. quadrivertex* , have long and strongly curved claws allowing them to attach firmly to hard substrates or plants and preventing them from being dislodged by waves during flooding (Pfingstl et al. 2020). Notably, *A. schneideri* , *H. pulchella* , and *Z. quadrivertex* are all sexual species, pointing to the dominance of sexual rather than parthenogenetic reproduction in salt marshes. Overall, this indicates that oribatid mite species in the PZ, but also the LSM and USM, are habitat and trophic specialists, explaining the dominance of deterministic factors in oribatid mite community assembly and the predictability of their community composition.

Similar to Oribatida, Mesostigmata communities differed strongly along the salt marsh transect. A total of 43% (15 of 35) of the Mesostigmata species only occurring in salt marsh habitats, including *Cheiroseius necorniger*, *C. salicorniae*, *Cyrthydrolaelaps incisus*, *Dendrolaelaps halophilus*, *Gamasodes fimbriatus*, *Halolaelaps fimbriatus*, *H. incisus* , *H. nodosus* , *H. strenzkei*, *Lysigamasus celticus*, *Pseudoparasitus dentatus*, *Rhodacarus salaries*, and *Vulgarogamasus trouessarti* (all Gamasina), and *Uropoda littoralis* and *Uropoda repleta* (Uropodina) (Table S3). Similar to Oribatida, a number of Mesostigmata species of the LSM and PZ possess a plastron as shown for *Cheiroseius* species (Hinton 1971) and several species of the genus *Uropoda* (Krantz 1974). Compared to oribatid mites, however, they may be less trophically specialized (Díaz‐Aguilar and Quideau 2013; Haynert et al. 2017), but this needs further investigation. Similar to Oribatida, most Mesostigmata species of the LSM and PZ reproduce sexually, but *Uropoda orbicularis* and *Rhodacarellus silesiacus* are likely to be parthenogenetic (Bloszyk et al. 2004; Walter and Oliver 1989). Overall, due to the large number of Mesostigmata species specializing in coastal living in the LSM and PZ which inhabit narrow niches (see also Haque et al. 2024), Mesostigmata communities in salt marshes are highly predictable and primarily structured by deterministic factors.

Collembola communities also included a number of species that only occur in coastal or littoral habitats, including *Anurida maritima* , *Archisotoma* spp., *Ballistura schoetti*, *Folsomia thallassophila*, *Halisotoma maritima*, *Proisotoma admaritima*, *Thalassaphorura* spp., *Xenylla acauda* , and *Xenylla tullbergi* (Table S1). These species occurred in each of the three salt marsh zones and were not restricted to the LSM or PZ, and this contributed to the less pronounced separation of Collembola communities along the salt marsh transect. Moreover, in contrast to Oribatida and Mesostigmata, several generalistic taxa, such as *Mesaphorura* spp. and *Paratullbergia* spp., occurred frequently in the PZ indicating that also generalist Collembola species are able to survive the harsh conditions in the PZ (Junggebauer et al. 2025). Similar to Oribatida and Mesostigmata, certain Collembola species are able to respire via plastron respiration, for example, *Anurida maritima* (King et al. 1990; Gundersen et al. 2014), and a number of other Collembola species are generally able to continue to respire even when submerged (Krzysztofowicz et al. 1972). Further, sea shore Collembola such as *Anurida maritima* tolerate high salinity or even require it for survival (Witteveen et al. 1987). The trophic ecology of sea shore Collembola species is not well studied but—according to stable isotope data—some species may be specialized algal feeders, such as *Halisotoma maritima*, *Isotoma viridis* , *Isotoma riparia*, and *Thalassaphorura debilis* (Haynert et al. 2017), and algal feeding also has been observed in *Proisotoma admaritima* (Fjellberg 1991). Overall, there are also a number of Collembola species specialized for colonizing salt marsh habitats, but their adaptations appear to be less strong than in Oribatida and Mesostigmata as indicated by their ability to colonize each of the three salt marsh zones (Haque et al. 2024). Collembola communities along the studied salt marsh transect therefore presumably are less structured by deterministic factors than Oribatida and Mesostigmata. Further, comparing the sensitivity of Collembola, Oribatida, and Mesostigmata communities to differences in forest types, Wenglein et al. (2025) found Collembola and Mesostigmata to be the less sensitive than Oribatida.

### 4.2 Soil Microarthropod Communities on the Three Islands

Generally, in each of the three microarthropod communities, the difference between the three islands was lower than between the three salt marsh zones, which supports our second hypothesis and indicates that the assembly of each of the three microarthropod communities is predominantly due to niche‐based deterministic rather than stochastic processes. However, the diversity of Oribatida was slightly higher on Spiekeroog island than on Norderney and Wangerooge, and the density of Collembola was slightly higher on Norderney and Spiekeroog than on Wangerooge, potentially reflecting stochastic processes (Ojala and Huhta 2001; Jenkins et al. 2007; Haque et al. 2024). At least in part, these differences may be explained by the rather low densities of microarthropods in salt marshes, which intrinsically have a higher level of turnover than larger populations (Orrock and Watling 2010). Moreover, the higher density of Collembola on Norderney may be due to a higher amount of nutrients. Norderney is located in close proximity to the Ems estuary, which may result in more nutrients entering the salt marshes of Norderney compared to the other islands, since the currents and nutrients along the islands flow eastwards. Overall, however, our findings suggest that despite strong abiotic forcing soil microarthropod communities in salt marshes are structured predominantly by deterministic factors assembling communities in a predictable way along salt marsh gradients, with this gradually differing between the three microarthropod taxa studied.

## Author Contributions

**Xue Pan:** conceptualization (equal), data curation (equal), formal analysis (equal), funding acquisition (equal), investigation (supporting), methodology (equal), software (equal), visualization (equal), writing – original draft (equal), writing – review and editing (equal). **Lara Warnke:** data curation (equal), formal analysis (equal), investigation (equal), methodology (equal), visualization (equal), writing – original draft (equal), writing – review and editing (equal). **Lennart Zwolski:** data curation (equal), investigation (equal), writing – review and editing (equal). **Maria Rinke:** data curation (equal), investigation (equal). **Ting‐Wen Chen:** writing – review and editing (equal). **Stefan Scheu:** conceptualization (equal), funding acquisition (equal), project administration (equal), supervision (equal), writing – review and editing (equal). **Mark Maraun:** conceptualization (equal), data curation (equal), formal analysis (equal), investigation (equal), methodology (equal), supervision (equal), visualization (equal), writing – original draft (equal), writing – review and editing (equal).

## Conflicts of Interest

The authors declare no conflicts of interest.

## Supporting information


**Table S1:** List of all Collembola collected along the salt marsh transect (LSM, lower salt marsh; PZ, pioneer zone; USM, upper salt marsh).
**Table S2:** List of all Oribatida collected along the salt marsh transect (LSM, lower salt marsh; PZ, pioneer zone; USM, upper salt marsh).
**Table S3:** List of all Mesostigmata collected along the salt marsh transect (LSM, lower salt marsh; PZ, pioneer zone; USM, upper salt marsh).

**Figure S1:** Rarefaction (species accumulation) curves for mesofauna taxa (Collembola, Oribatida, and Mesostigmata combined) in (a) the upper salt marsh (USM), lower salt marsh (LSM) and pioneer zone (PZ), and (b) on the three islands studied. Dotted lines show extrapolated species numbers. Colored areas represent the variance of the respective curve.
**Figure S2:** Density of (a) Collembola on the three islands and (b) in the upper salt marsh, lower salt marsh and pioneer zone, and (c) Oribatida in the three salt marsh zones on each of the three islands studied (means ± SD). For statistical analyses see text.
**Figure S3:** Species‐level dissimilarity matrix heat maps along the salt marsh zones (a) and between the three islands for Collembola, Oribatida, and Mesostigmata together. The Bray–Curtis dissimilarity (*β*_bc_) is partitioned into (i) the turnover component (*β*_bc.bal_) and (ii) the nestedness component (*β*_bc.gra_). Dendrograms are based on hierarchical cluster analysis (unweighted pair group method with arithmetic averages; UPGMA) of the dissimilarity matrix.
**Figure S4:** Community composition of Collembola in the upper salt marsh (USM), lower salt marsh (LSM), and pioneer zone (PZ) on the islands Norderney, Spiekeroog, and Wangerooge as indicated by the first two axes of nonmetric multidimensional scaling (NMDS).
**Figure S5:** Community composition of Mesostigmata in the upper salt marsh (USM), lower salt marsh (LSM) and pioneer zone (PZ) on the islands Norderney, Spiekeroog, and Wangerooge as indicated by the first two axes of nonmetric multidimensional scaling (NMDS).
**Figure S6:** Community composition of Oribatida in the upper salt marsh (USM), lower salt marsh (LSM) and pioneer zone (PZ) on the islands Norderney, Spiekeroog, and Wangerooge as indicated by the first two axes of nonmetric multidimensional scaling (NMDS).
**Figure S7:** Venn diagrams of all microarthropod taxa (Mesostigmata, Oribatida, Collembola) in (a) the salt marsh zone and (b) on the three islands.

## Data Availability

All data generated or analyzed during this study are available in the Supporting Information of this article and are available from an open digital repository (Dryad, https://doi.org/10.5061/dryad.xd2547dw7).
