# Supplementary material for: Soil Microarthropod Communities Along Salt Marsh Transects of the Wadden Sea Are Predominantly Structured by Niche Differentiation
Source: Ecol Evol. 2026 Mar 20;16(3):e73245. doi: 10.1002/ece3.73245 (PMC13093831; doi:10.1002/ece3.73245)
Supplement: Supplementary file 1 — Table S1: List of all Collembola collected along the salt marsh transect (LSM, lower salt marsh; PZ, pioneer zone; USM, upper salt marsh). Table S2: List of all Oribatida collected along the salt marsh transect (LSM, lower salt marsh; PZ, pioneer zone; USM, upper salt marsh). Table S3: List of all Mesostigmata collected along the salt marsh transect (LSM, lower salt marsh; PZ, pioneer zone; USM, upper salt marsh). [file ECE3-16-e73245-s001.zip › 005_Appendix Table 1 Collembola.docx]

| **Table 1.** List of all Collembola collected along the salt marsh transect (USM = upper salt marsh, LSM = lower salt marsh, PZ = pioneer zone) | | | | | | | | |  |  |  |  |  |  |  |  |  |  |  |  |  |  |  |  |  |  |  |  |  |  |  |  |
| --- | --- | --- | --- | --- | --- | --- | --- | --- | --- | --- | --- | --- | --- | --- | --- | --- | --- | --- | --- | --- | --- | --- | --- | --- | --- | --- | --- | --- | --- | --- | --- | --- |
| of the three islands (Norderney, Wangerooge, Spiekeroog) | | | |  |  |  |  |  |  |  |  |  |  |  |  |  |  |  |  |  |  |  |  |  |  |  |  |  |  |  |  |  |
|  |  |  |  |  |  |  |  |  |  |  |  |  |  |  |  |  |  |  |  |  |  |  |  |  |  |  |  |  |  |  |  |  |
| **Insel** | **Transekt** | Anurida maritima | Archisotoma besselsi | Archisotoma megalops | Archisotoma sp. | Archisotoma theae | Ballistura schoetti | Cryptopygus thermiphilis | Entomobrya marginate | Frisea cliviseta | Frisea mirabilis | Frisea truncata | Folsomia sexoculata | Folsomia thalassophila | Halisotoma maritima | Hypogastrura manubrialis | Hypogastrura sahlbergi | Isotoma viridis | Isotomella minor | Lepidocyrtus lanuginosus | Mesaphorura sp. | Paratullbergia sp. | Parisotoma notabilis | Proisotoma admaritima | Pseudisotoma sensibilis | Sphaeridia pumilis | Stenaphorura denisi | Thalassaphorura debilis | Thalassaphorura halophila | Willemia sp. | Xenylla acauda | Xenylla tullbergi |
| Norderney | LSM | 0 | 0 | 0 | 0 | 0 | 0 | 1 | 0 | 6 | 2 | 2 | 0 | 0 | 0 | 0 | 0 | 0 | 0 | 0 | 61 | 69 | 7 | 0 | 0 | 0 | 0 | 21 | 22 | 0 | 0 | 0 |
| Norderney | LSM | 0 | 0 | 0 | 0 | 0 | 0 | 0 | 0 | 1 | 0 | 0 | 0 | 0 | 0 | 0 | 0 | 1 | 0 | 0 | 47 | 41 | 1 | 0 | 1 | 0 | 0 | 26 | 6 | 2 | 0 | 0 |
| Norderney | LSM | 0 | 0 | 2 | 0 | 0 | 0 | 0 | 0 | 0 | 3 | 1 | 0 | 0 | 0 | 0 | 0 | 0 | 0 | 0 | 32 | 40 | 1 | 0 | 1 | 0 | 0 | 28 | 20 | 2 | 0 | 0 |
| Norderney | LSM | 0 | 0 | 0 | 0 | 0 | 0 | 0 | 0 | 0 | 0 | 3 | 0 | 0 | 0 | 0 | 0 | 0 | 0 | 0 | 14 | 15 | 2 | 0 | 0 | 0 | 0 | 10 | 7 | 0 | 0 | 0 |
| Norderney | LSM | 0 | 0 | 0 | 0 | 0 | 0 | 0 | 0 | 0 | 0 | 0 | 0 | 0 | 0 | 0 | 0 | 0 | 0 | 0 | 9 | 0 | 0 | 0 | 0 | 0 | 0 | 1 | 0 | 0 | 0 | 0 |
| Norderney | PZ | 0 | 0 | 4 | 0 | 0 | 0 | 0 | 0 | 0 | 0 | 0 | 0 | 0 | 0 | 0 | 0 | 0 | 0 | 0 | 0 | 0 | 0 | 0 | 0 | 0 | 0 | 6 | 1 | 0 | 0 | 0 |
| Norderney | PZ | 0 | 0 | 1 | 0 | 0 | 0 | 0 | 0 | 0 | 0 | 0 | 0 | 0 | 0 | 0 | 0 | 0 | 0 | 0 | 0 | 0 | 0 | 2 | 0 | 0 | 0 | 0 | 1 | 0 | 0 | 0 |
| Norderney | PZ | 0 | 0 | 0 | 0 | 0 | 0 | 0 | 0 | 0 | 0 | 0 | 0 | 0 | 0 | 0 | 0 | 0 | 0 | 0 | 0 | 1 | 0 | 0 | 0 | 0 | 0 | 1 | 0 | 0 | 0 | 0 |
| Norderney | PZ | 0 | 0 | 0 | 0 | 0 | 0 | 0 | 0 | 0 | 0 | 0 | 0 | 0 | 0 | 0 | 0 | 0 | 0 | 0 | 0 | 4 | 0 | 0 | 0 | 0 | 0 | 3 | 2 | 0 | 0 | 0 |
| Norderney | PZ | 0 | 0 | 0 | 0 | 0 | 0 | 0 | 0 | 0 | 1 | 0 | 0 | 0 | 0 | 0 | 0 | 0 | 0 | 0 | 56 | 19 | 0 | 0 | 0 | 0 | 0 | 6 | 1 | 0 | 0 | 0 |
| Norderney | USM | 0 | 0 | 0 | 0 | 0 | 0 | 0 | 0 | 0 | 0 | 0 | 0 | 0 | 0 | 0 | 0 | 3 | 0 | 0 | 2 | 0 | 0 | 0 | 0 | 0 | 0 | 8 | 1 | 0 | 0 | 0 |
| Norderney | USM | 0 | 0 | 0 | 0 | 0 | 5 | 1 | 0 | 2 | 0 | 1 | 0 | 0 | 0 | 0 | 0 | 2 | 0 | 0 | 5 | 22 | 0 | 0 | 0 | 0 | 0 | 12 | 4 | 0 | 0 | 0 |
| Norderney | USM | 0 | 1 | 0 | 0 | 0 | 0 | 1 | 0 | 0 | 0 | 0 | 0 | 0 | 0 | 0 | 0 | 3 | 0 | 0 | 14 | 16 | 0 | 0 | 0 | 0 | 0 | 10 | 0 | 0 | 0 | 0 |
| Norderney | USM | 0 | 0 | 0 | 1 | 0 | 0 | 0 | 0 | 0 | 0 | 1 | 1 | 1 | 5 | 0 | 0 | 2 | 0 | 0 | 21 | 16 | 0 | 0 | 0 | 0 | 1 | 6 | 3 | 0 | 0 | 0 |
| Norderney | USM | 0 | 0 | 0 | 0 | 0 | 0 | 5 | 0 | 0 | 0 | 0 | 0 | 0 | 10 | 0 | 0 | 0 | 0 | 0 | 21 | 0 | 0 | 0 | 1 | 0 | 0 | 5 | 3 | 0 | 0 | 0 |
| Spiekeroog | LSM | 0 | 0 | 0 | 0 | 0 | 0 | 0 | 0 | 0 | 0 | 0 | 0 | 0 | 0 | 0 | 0 | 0 | 0 | 0 | 25 | 27 | 0 | 0 | 0 | 0 | 0 | 8 | 4 | 0 | 0 | 0 |
| Spiekeroog | LSM | 0 | 1 | 1 | 0 | 0 | 0 | 0 | 0 | 0 | 0 | 0 | 0 | 0 | 0 | 0 | 0 | 0 | 0 | 0 | 42 | 38 | 0 | 0 | 0 | 0 | 0 | 34 | 18 | 0 | 0 | 0 |
| Spiekeroog | LSM | 0 | 0 | 0 | 0 | 0 | 0 | 0 | 0 | 0 | 0 | 0 | 0 | 0 | 0 | 0 | 0 | 0 | 0 | 0 | 14 | 9 | 0 | 0 | 0 | 0 | 0 | 4 | 10 | 0 | 0 | 0 |
| Spiekeroog | LSM | 0 | 0 | 0 | 0 | 0 | 0 | 0 | 0 | 0 | 0 | 0 | 0 | 0 | 0 | 0 | 0 | 0 | 0 | 0 | 23 | 15 | 0 | 0 | 0 | 0 | 0 | 1 | 3 | 0 | 0 | 0 |
| Spiekeroog | LSM | 0 | 0 | 0 | 0 | 0 | 0 | 0 | 0 | 0 | 0 | 0 | 0 | 0 | 0 | 0 | 0 | 0 | 0 | 0 | 11 | 0 | 0 | 0 | 0 | 0 | 0 | 1 | 2 | 0 | 0 | 0 |
| Spiekeroog | PZ | 0 | 0 | 22 | 0 | 0 | 0 | 0 | 0 | 0 | 0 | 0 | 0 | 0 | 0 | 0 | 0 | 0 | 0 | 1 | 2 | 0 | 0 | 0 | 0 | 0 | 0 | 3 | 0 | 0 | 0 | 0 |
| Spiekeroog | PZ | 0 | 0 | 24 | 0 | 0 | 0 | 0 | 0 | 0 | 0 | 0 | 0 | 0 | 0 | 0 | 0 | 0 | 1 | 0 | 3 | 1 | 1 | 0 | 0 | 0 | 0 | 0 | 0 | 0 | 0 | 0 |
| Spiekeroog | PZ | 0 | 0 | 1 | 0 | 0 | 0 | 0 | 0 | 1 | 0 | 0 | 0 | 0 | 0 | 0 | 0 | 0 | 0 | 0 | 0 | 0 | 0 | 0 | 0 | 0 | 0 | 11 | 0 | 0 | 0 | 0 |
| Spiekeroog | USM | 0 | 0 | 0 | 0 | 0 | 0 | 0 | 0 | 0 | 0 | 0 | 0 | 0 | 0 | 0 | 0 | 0 | 0 | 0 | 13 | 5 | 2 | 0 | 0 | 0 | 0 | 0 | 0 | 0 | 0 | 0 |
| Spiekeroog | USM | 0 | 0 | 0 | 0 | 0 | 0 | 0 | 0 | 0 | 0 | 0 | 0 | 0 | 0 | 0 | 0 | 0 | 0 | 0 | 34 | 2 | 0 | 0 | 0 | 0 | 0 | 0 | 0 | 0 | 0 | 0 |
| Spiekeroog | USM | 0 | 0 | 7 | 0 | 0 | 0 | 0 | 1 | 0 | 1 | 1 | 0 | 0 | 2 | 0 | 0 | 1 | 0 | 0 | 102 | 14 | 3 | 1 | 9 | 2 | 0 | 0 | 0 | 0 | 2 | 0 |
| Spiekeroog | USM | 0 | 0 | 0 | 0 | 0 | 0 | 0 | 0 | 0 | 1 | 1 | 0 | 0 | 0 | 0 | 0 | 0 | 0 | 0 | 28 | 5 | 6 | 0 | 0 | 0 | 0 | 5 | 3 | 0 | 0 | 0 |
| Spiekeroog | USM | 1 | 0 | 0 | 0 | 0 | 0 | 0 | 0 | 0 | 0 | 2 | 0 | 0 | 4 | 0 | 0 | 0 | 0 | 0 | 66 | 9 | 3 | 1 | 0 | 0 | 0 | 2 | 0 | 0 | 1 | 0 |
| Wangerooge | LSM | 0 | 0 | 0 | 0 | 0 | 0 | 0 | 0 | 0 | 0 | 1 | 0 | 0 | 0 | 0 | 0 | 0 | 0 | 0 | 3 | 2 | 0 | 0 | 0 | 0 | 0 | 1 | 0 | 0 | 0 | 0 |
| Wangerooge | LSM | 0 | 0 | 0 | 0 | 0 | 0 | 0 | 0 | 0 | 0 | 0 | 0 | 0 | 0 | 0 | 0 | 0 | 0 | 0 | 1 | 0 | 0 | 0 | 0 | 0 | 0 | 0 | 0 | 0 | 0 | 0 |
| Wangerooge | LSM | 0 | 0 | 1 | 0 | 1 | 0 | 0 | 0 | 0 | 1 | 0 | 2 | 0 | 0 | 0 | 0 | 0 | 0 | 0 | 1 | 5 | 0 | 1 | 0 | 0 | 0 | 0 | 0 | 0 | 0 | 0 |
| Wangerooge | PZ | 0 | 0 | 0 | 0 | 0 | 0 | 0 | 0 | 0 | 0 | 0 | 0 | 0 | 0 | 0 | 0 | 0 | 0 | 0 | 1 | 0 | 0 | 0 | 0 | 0 | 0 | 0 | 0 | 0 | 0 | 0 |
| Wangerooge | PZ | 0 | 0 | 0 | 0 | 0 | 0 | 0 | 0 | 0 | 0 | 0 | 0 | 0 | 0 | 0 | 0 | 0 | 0 | 0 | 5 | 0 | 0 | 1 | 0 | 0 | 0 | 0 | 0 | 0 | 0 | 0 |
| Wangerooge | PZ | 0 | 0 | 0 | 0 | 0 | 0 | 0 | 0 | 0 | 0 | 0 | 0 | 0 | 0 | 0 | 0 | 0 | 0 | 0 | 12 | 2 | 0 | 0 | 0 | 0 | 0 | 0 | 0 | 0 | 0 | 0 |
| Wangerooge | PZ | 0 | 0 | 0 | 0 | 0 | 0 | 0 | 0 | 0 | 0 | 0 | 0 | 0 | 0 | 0 | 0 | 0 | 0 | 0 | 3 | 0 | 0 | 0 | 0 | 0 | 0 | 0 | 0 | 0 | 0 | 0 |
| Wangerooge | USM | 0 | 0 | 1 | 0 | 1 | 0 | 0 | 0 | 0 | 0 | 0 | 7 | 1 | 0 | 0 | 0 | 0 | 0 | 0 | 4 | 4 | 1 | 0 | 0 | 0 | 0 | 3 | 0 | 0 | 0 | 2 |
| Wangerooge | USM | 0 | 0 | 21 | 3 | 2 | 0 | 0 | 0 | 1 | 0 | 0 | 0 | 0 | 0 | 11 | 1 | 0 | 0 | 0 | 39 | 18 | 0 | 0 | 0 | 0 | 1 | 5 | 1 | 0 | 1 | 0 |
| Wangerooge | USM | 0 | 0 | 13 | 0 | 1 | 0 | 0 | 0 | 0 | 0 | 0 | 0 | 0 | 0 | 2 | 2 | 0 | 0 | 0 | 3 | 2 | 0 | 0 | 0 | 0 | 0 | 1 | 2 | 0 | 0 | 0 |
| Wangerooge | USM | 0 | 0 | 0 | 0 | 1 | 0 | 0 | 0 | 0 | 0 | 0 | 0 | 0 | 0 | 0 | 0 | 0 | 0 | 0 | 5 | 1 | 0 | 0 | 0 | 0 | 0 | 0 | 0 | 0 | 0 | 0 |
| Wangerooge | USM | 0 | 0 | 5 | 0 | 0 | 0 | 0 | 0 | 0 | 0 | 0 | 0 | 0 | 0 | 1 | 0 | 0 | 0 | 0 | 5 | 8 | 1 | 1 | 0 | 0 | 0 | 2 | 0 | 0 | 0 | 0 |
|  |  |  |  |  |  |  |  |  |  |  |  |  |  |  |  |  |  |  |  |  |  |  |  |  |  |  |  |  |  |  |  |  |
