# Supplementary material for: Soil Microarthropod Communities Along Salt Marsh Transects of the Wadden Sea Are Predominantly Structured by Niche Differentiation
Source: Ecol Evol. 2026 Mar 20;16(3):e73245. doi: 10.1002/ece3.73245 (PMC13093831; doi:10.1002/ece3.73245)
Supplement: Supplementary file 1 — Table S1: List of all Collembola collected along the salt marsh transect (LSM, lower salt marsh; PZ, pioneer zone; USM, upper salt marsh). Table S2: List of all Oribatida collected along the salt marsh transect (LSM, lower salt marsh; PZ, pioneer zone; USM, upper salt marsh). Table S3: List of all Mesostigmata collected along the salt marsh transect (LSM, lower salt marsh; PZ, pioneer zone; USM, upper salt marsh). [file ECE3-16-e73245-s001.zip › 006_Appendix Table 2 Oribatida.docx]

| **Table 2.** List of all Oribatida collected along the salt marsh transect (USM = upper salt marsh, LSM = lower salt marsh, PZ = pioneer zone) | | | | | |  |  |  |  |  |  |  |  |  |  |  |  |
| --- | --- | --- | --- | --- | --- | --- | --- | --- | --- | --- | --- | --- | --- | --- | --- | --- | --- |
| of the three islands (Norderney, Wangerooge, Spiekeroog) | | | |  |  |  |  |  |  |  |  |  |  |  |  |  |  |
|  |  |  |  |  |  |  |  |  |  |  |  |  |  |  |  |  |  |
| Insel | Transekt | Ameronothrus schneideri | Ameronothrus juv. | Banksinoma lanceolata | Cultroribula bicultrata | Hermannia pulchella | Hermannia juv. | Liebstadia similis | Metabelba pulverosa | Microppia minus | Oppiella neerlandica | Oribatida juv. indet. | Ramusella clavipectinata | Suctobelbella subcornigera | Tectocepheus sarkensis | Trichoribates incisellus | Zachvatkinibates quadrivertex |
| Norderney | LSM | 0 | 0 | 0 | 0 | 0 | 0 | 0 | 1 | 0 | 4 | 0 | 0 | 0 | 0 | 0 | 1 |
| Norderney | LSM | 0 | 0 | 0 | 0 | 0 | 0 | 0 | 0 | 0 | 2 | 0 | 0 | 0 | 0 | 0 | 4 |
| Norderney | LSM | 0 | 0 | 0 | 0 | 0 | 0 | 0 | 0 | 0 | 4 | 0 | 0 | 0 | 0 | 0 | 8 |
| Norderney | LSM | 0 | 0 | 0 | 0 | 0 | 0 | 0 | 0 | 0 | 0 | 0 | 0 | 0 | 0 | 0 | 0 |
| Norderney | LSM | 0 | 0 | 0 | 0 | 0 | 0 | 0 | 0 | 0 | 2 | 0 | 0 | 0 | 0 | 0 | 2 |
| Norderney | PZ | 0 | 0 | 0 | 0 | 0 | 0 | 0 | 0 | 0 | 0 | 0 | 0 | 0 | 0 | 0 | 1 |
| Norderney | PZ | 0 | 0 | 0 | 0 | 0 | 0 | 0 | 0 | 0 | 0 | 0 | 0 | 0 | 0 | 0 | 0 |
| Norderney | PZ | 0 | 0 | 0 | 0 | 0 | 0 | 0 | 0 | 0 | 0 | 0 | 0 | 1 | 0 | 0 | 0 |
| Norderney | PZ | 0 | 0 | 0 | 0 | 0 | 0 | 0 | 0 | 0 | 0 | 0 | 0 | 0 | 0 | 0 | 0 |
| Norderney | PZ | 0 | 0 | 0 | 0 | 0 | 0 | 0 | 0 | 0 | 1 | 0 | 0 | 0 | 0 | 0 | 0 |
| Norderney | USM | 0 | 0 | 0 | 0 | 0 | 0 | 0 | 0 | 0 | 0 | 0 | 6 | 0 | 0 | 3 | 0 |
| Norderney | USM | 0 | 0 | 0 | 0 | 0 | 0 | 1 | 0 | 0 | 0 | 0 | 0 | 0 | 0 | 0 | 0 |
| Norderney | USM | 0 | 0 | 0 | 0 | 0 | 0 | 1 | 0 | 0 | 0 | 0 | 1 | 0 | 0 | 0 | 0 |
| Norderney | USM | 0 | 0 | 0 | 0 | 0 | 1 | 0 | 0 | 0 | 0 | 0 | 0 | 0 | 0 | 1 | 0 |
| Norderney | USM | 0 | 0 | 0 | 0 | 0 | 0 | 0 | 0 | 0 | 0 | 0 | 0 | 0 | 0 | 0 | 0 |
| Spiekeroog | LSM | 0 | 0 | 0 | 0 | 0 | 3 | 0 | 0 | 0 | 0 | 0 | 0 | 0 | 0 | 0 | 3 |
| Spiekeroog | LSM | 0 | 0 | 0 | 0 | 1 | 1 | 0 | 0 | 0 | 0 | 0 | 0 | 0 | 0 | 0 | 0 |
| Spiekeroog | LSM | 0 | 0 | 0 | 0 | 7 | 21 | 0 | 0 | 0 | 1 | 0 | 0 | 0 | 0 | 0 | 3 |
| Spiekeroog | LSM | 0 | 0 | 0 | 0 | 3 | 0 | 0 | 0 | 0 | 0 | 0 | 0 | 0 | 0 | 0 | 4 |
| Spiekeroog | LSM | 0 | 1 | 0 | 1 | 0 | 0 | 0 | 0 | 0 | 0 | 0 | 0 | 0 | 0 | 0 | 0 |
| Spiekeroog | PZ | 0 | 0 | 0 | 0 | 0 | 0 | 0 | 0 | 0 | 0 | 0 | 0 | 0 | 0 | 0 | 2 |
| Spiekeroog | PZ | 0 | 0 | 0 | 0 | 0 | 0 | 0 | 0 | 0 | 0 | 0 | 0 | 0 | 0 | 0 | 1 |
| Spiekeroog | PZ | 0 | 0 | 0 | 0 | 0 | 0 | 0 | 0 | 0 | 0 | 1 | 0 | 0 | 0 | 0 | 0 |
| Spiekeroog | PZ | 0 | 0 | 0 | 0 | 0 | 0 | 0 | 0 | 0 | 0 | 2 | 0 | 0 | 0 | 0 | 0 |
| Spiekeroog | PZ | 0 | 0 | 0 | 0 | 0 | 0 | 0 | 0 | 0 | 0 | 0 | 0 | 0 | 0 | 0 | 1 |
| Spiekeroog | USM | 0 | 0 | 2 | 0 | 0 | 0 | 1 | 0 | 0 | 0 | 0 | 25 | 0 | 1 | 0 | 0 |
| Spiekeroog | USM | 0 | 0 | 0 | 0 | 0 | 0 | 0 | 0 | 1 | 0 | 0 | 10 | 0 | 0 | 0 | 0 |
| Spiekeroog | USM | 0 | 0 | 3 | 0 | 0 | 0 | 2 | 0 | 23 | 0 | 0 | 24 | 0 | 1 | 0 | 0 |
| Spiekeroog | USM | 0 | 0 | 0 | 0 | 0 | 0 | 0 | 0 | 52 | 0 | 0 | 3 | 0 | 3 | 0 | 0 |
| Spiekeroog | USM | 0 | 0 | 0 | 0 | 0 | 0 | 0 | 0 | 11 | 0 | 1 | 2 | 0 | 8 | 0 | 0 |
| Wangerooge | LSM | 0 | 0 | 0 | 0 | 0 | 0 | 0 | 0 | 0 | 0 | 0 | 0 | 0 | 0 | 0 | 0 |
| Wangerooge | LSM | 0 | 1 | 0 | 0 | 0 | 0 | 0 | 0 | 0 | 0 | 0 | 0 | 0 | 0 | 0 | 0 |
| Wangerooge | LSM | 0 | 0 | 0 | 0 | 9 | 3 | 0 | 0 | 0 | 0 | 0 | 0 | 0 | 0 | 0 | 2 |
| Wangerooge | LSM | 1 | 2 | 0 | 0 | 2 | 6 | 0 | 0 | 0 | 0 | 0 | 0 | 0 | 0 | 0 | 0 |
| Wangerooge | LSM | 0 | 1 | 0 | 0 | 0 | 0 | 0 | 0 | 0 | 0 | 0 | 0 | 0 | 0 | 0 | 0 |
| Wangerooge | PZ | 0 | 0 | 0 | 0 | 0 | 0 | 0 | 0 | 0 | 0 | 0 | 0 | 0 | 0 | 0 | 0 |
| Wangerooge | PZ | 0 | 0 | 0 | 0 | 0 | 0 | 0 | 0 | 0 | 0 | 1 | 0 | 0 | 0 | 0 | 0 |
| Wangerooge | PZ | 0 | 0 | 0 | 0 | 0 | 0 | 0 | 0 | 0 | 0 | 0 | 0 | 0 | 0 | 0 | 0 |
| Wangerooge | PZ | 0 | 1 | 0 | 0 | 0 | 0 | 0 | 0 | 0 | 0 | 0 | 0 | 0 | 0 | 0 | 0 |
| Wangerooge | PZ | 0 | 0 | 0 | 0 | 0 | 0 | 0 | 0 | 0 | 0 | 0 | 0 | 0 | 0 | 0 | 0 |
| Wangerooge | USM | 0 | 0 | 0 | 0 | 1 | 5 | 0 | 0 | 0 | 0 | 0 | 0 | 0 | 0 | 0 | 0 |
| Wangerooge | USM | 0 | 0 | 0 | 0 | 0 | 0 | 0 | 0 | 0 | 0 | 0 | 2 | 0 | 0 | 1 | 0 |
| Wangerooge | USM | 0 | 1 | 0 | 0 | 0 | 3 | 0 | 0 | 0 | 0 | 0 | 0 | 0 | 0 | 0 | 0 |
| Wangerooge | USM | 0 | 0 | 0 | 0 | 1 | 0 | 0 | 0 | 0 | 0 | 0 | 0 | 0 | 0 | 0 | 0 |
| Wangerooge | USM | 0 | 0 | 0 | 0 | 0 | 0 | 0 | 0 | 0 | 1 | 0 | 0 | 0 | 0 | 0 | 0 |
