# Supplementary material for: Soil Microarthropod Communities Along Salt Marsh Transects of the Wadden Sea Are Predominantly Structured by Niche Differentiation
Source: Ecol Evol. 2026 Mar 20;16(3):e73245. doi: 10.1002/ece3.73245 (PMC13093831; doi:10.1002/ece3.73245)
Supplement: Supplementary file 2 — Figure S1: Rarefaction (species accumulation) curves for mesofauna taxa (Collembola, Oribatida, and Mesostigmata combined) in (a) the upper salt marsh (USM), lower salt marsh (LSM) and pioneer zone (PZ), and (b) on the three islands studied. Dotted lines show extrapolated species numbers. Colored areas represent the variance of the respective curve. Figure S2: Density of (a) Collembola on the three islands and (b) in the upper salt marsh, lower salt marsh and pioneer zone, and (c) Oribatida in the three salt marsh zones on each of the three islands studied (means ± SD). For statistical analyses see text. Figure S3: Species‐level dissimilarity matrix heat maps along the salt marsh zones (a) and between the three islands for Collembola, Oribatida, and Mesostigmata together. The Bray–Curtis dissimilarity (β bc) is partitioned into (i) the turnover component (β bc.bal) and (ii) the nestedness component (β bc.gra). Dendrograms are based on hierarchical cluster analysis (unweighted pair group method with arithmetic averages; UPGMA) of the dissimilarity matrix. Figure S4: Community composition of Collembola in the upper salt marsh (USM), lower salt marsh (LSM), and pioneer zone (PZ) on the islands Norderney, Spiekeroog, and Wangerooge as indicated by the first two axes of nonmetric multidimensional scaling (NMDS). Figure S5: Community composition of Mesostigmata in the upper salt marsh (USM), lower salt marsh (LSM) and pioneer zone (PZ) on the islands Norderney, Spiekeroog, and Wangerooge as indicated by the first two axes of nonmetric multidimensional scaling (NMDS). Figure S6: Community composition of Oribatida in the upper salt marsh (USM), lower salt marsh (LSM) and pioneer zone (PZ) on the islands Norderney, Spiekeroog, and Wangerooge as indicated by the first two axes of nonmetric multidimensional scaling (NMDS). Figure S7: Venn diagrams of all microarthropod taxa (Mesostigmata, Oribatida, Collembola) in (a) the salt marsh zone and (b) on the three islands. [file ECE3-16-e73245-s002.pptx]

## Slide 1
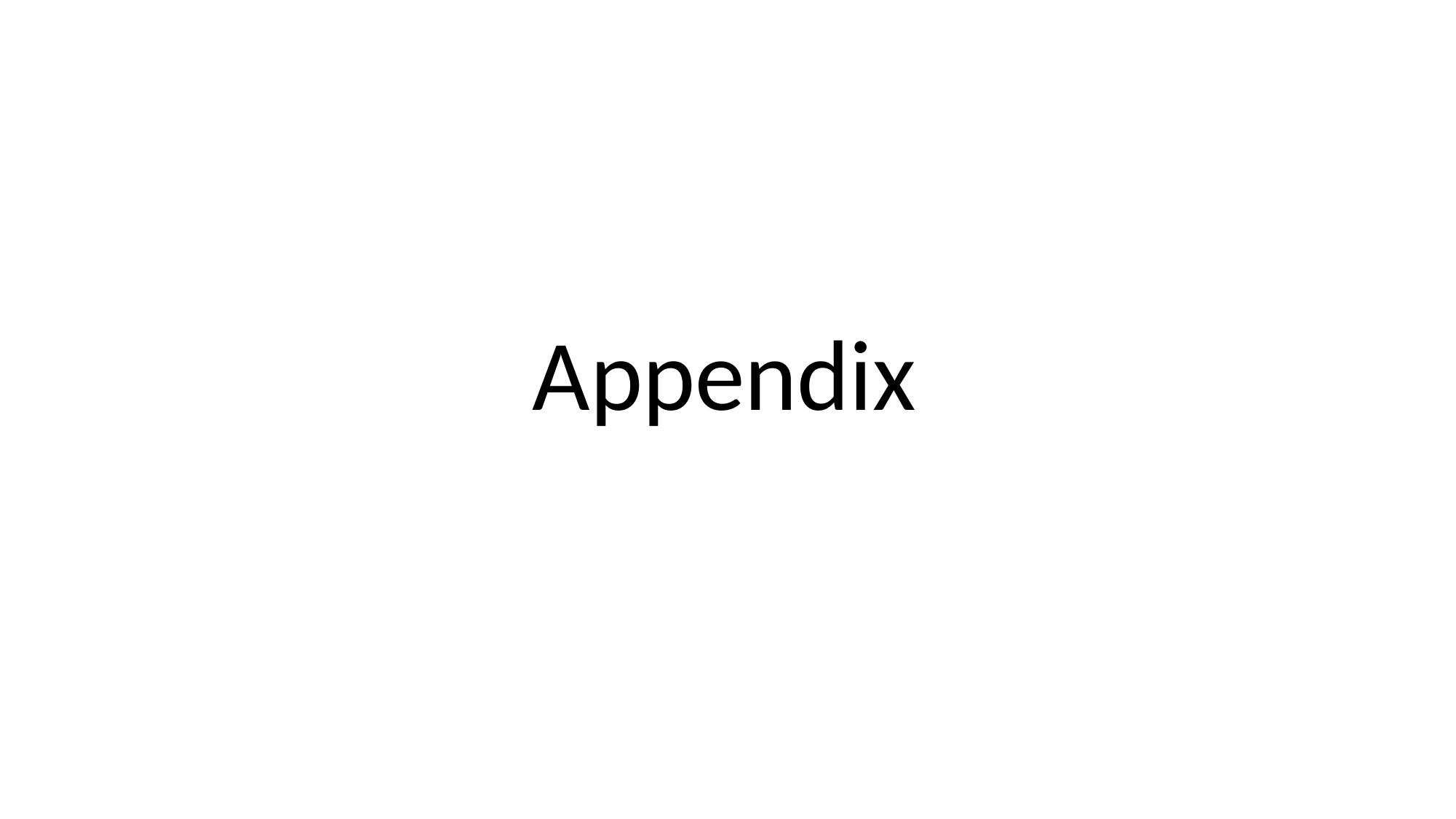

Appendix

## Slide 2
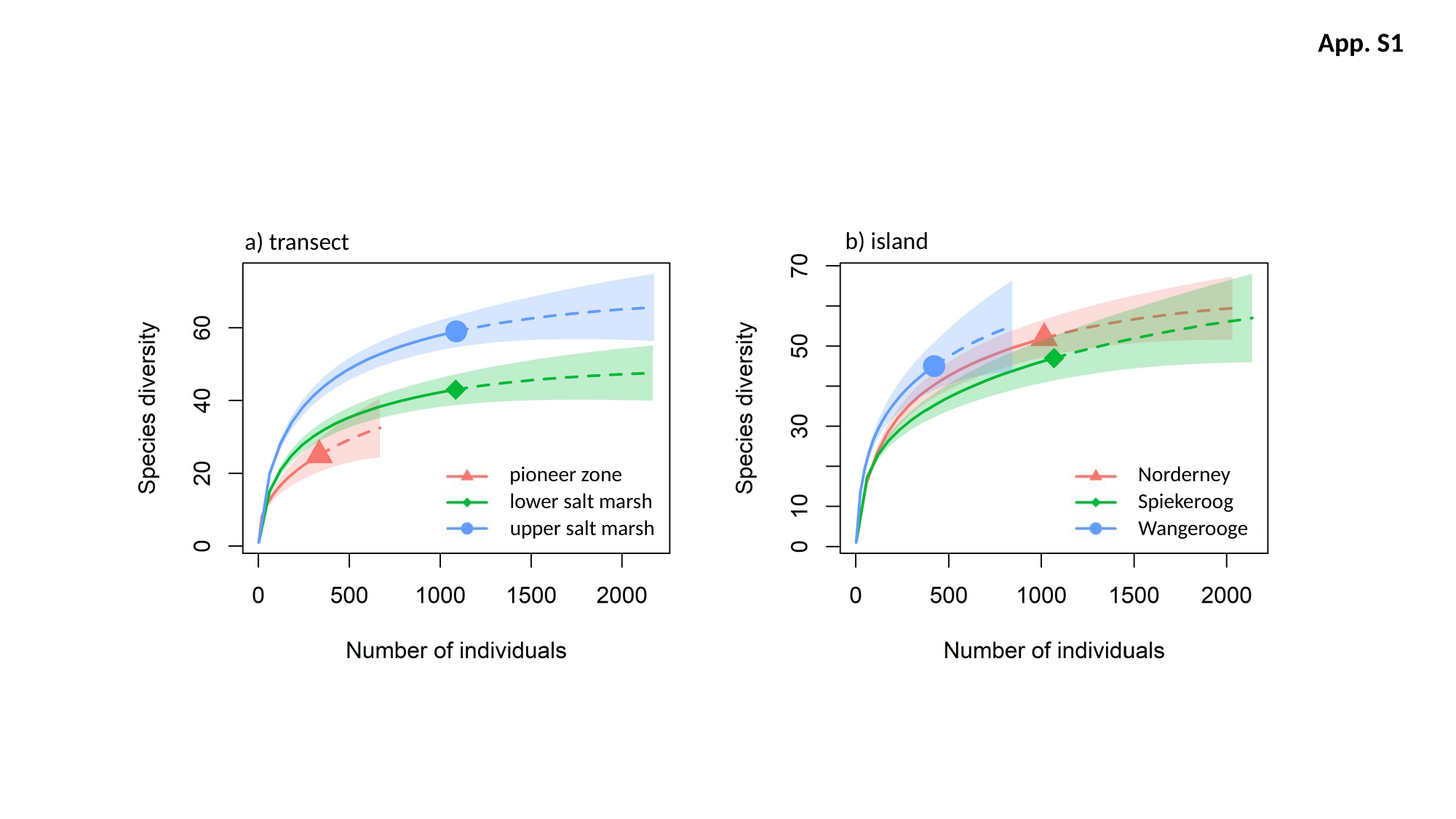

App. S1
b) island
a) transect
pioneer zone
lower salt marsh
upper salt marsh
Norderney
Spiekeroog
Wangerooge

## Slide 3
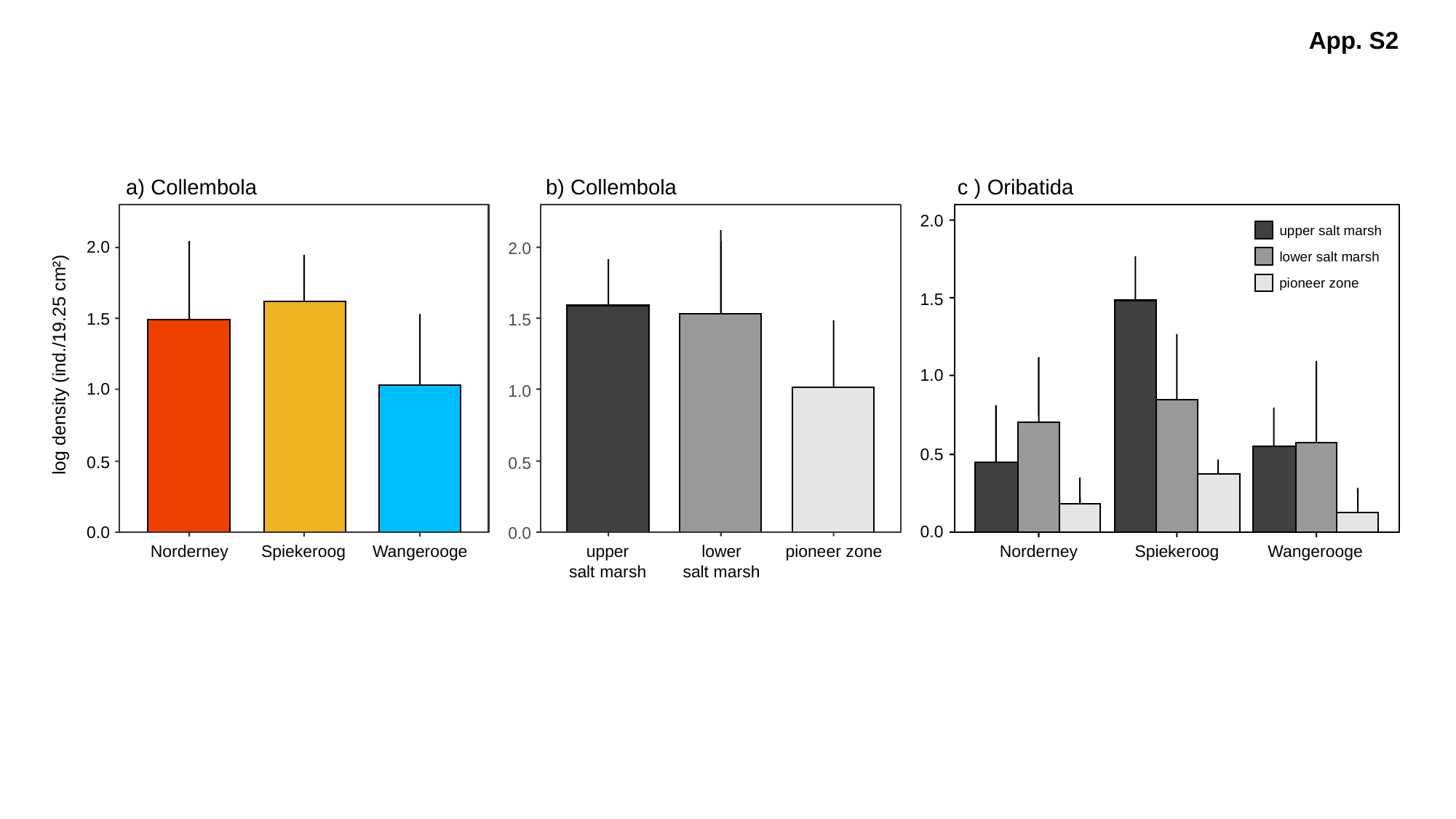

App. S2
a) Collembola
b) Collembola
c ) Oribatida
2.0
upper salt marsh
2.0
2.0
lower salt marsh
pioneer zone
1.5
1.5
1.5
log density (ind./19.25 cm²)
1.0
1.0
1.0
0.5
0.5
0.5
0.0
0.0
0.0
Norderney
Spiekeroog
Wangerooge
upper
salt marsh
lower
salt marsh
pioneer zone
Norderney
Spiekeroog
Wangerooge

## Slide 4
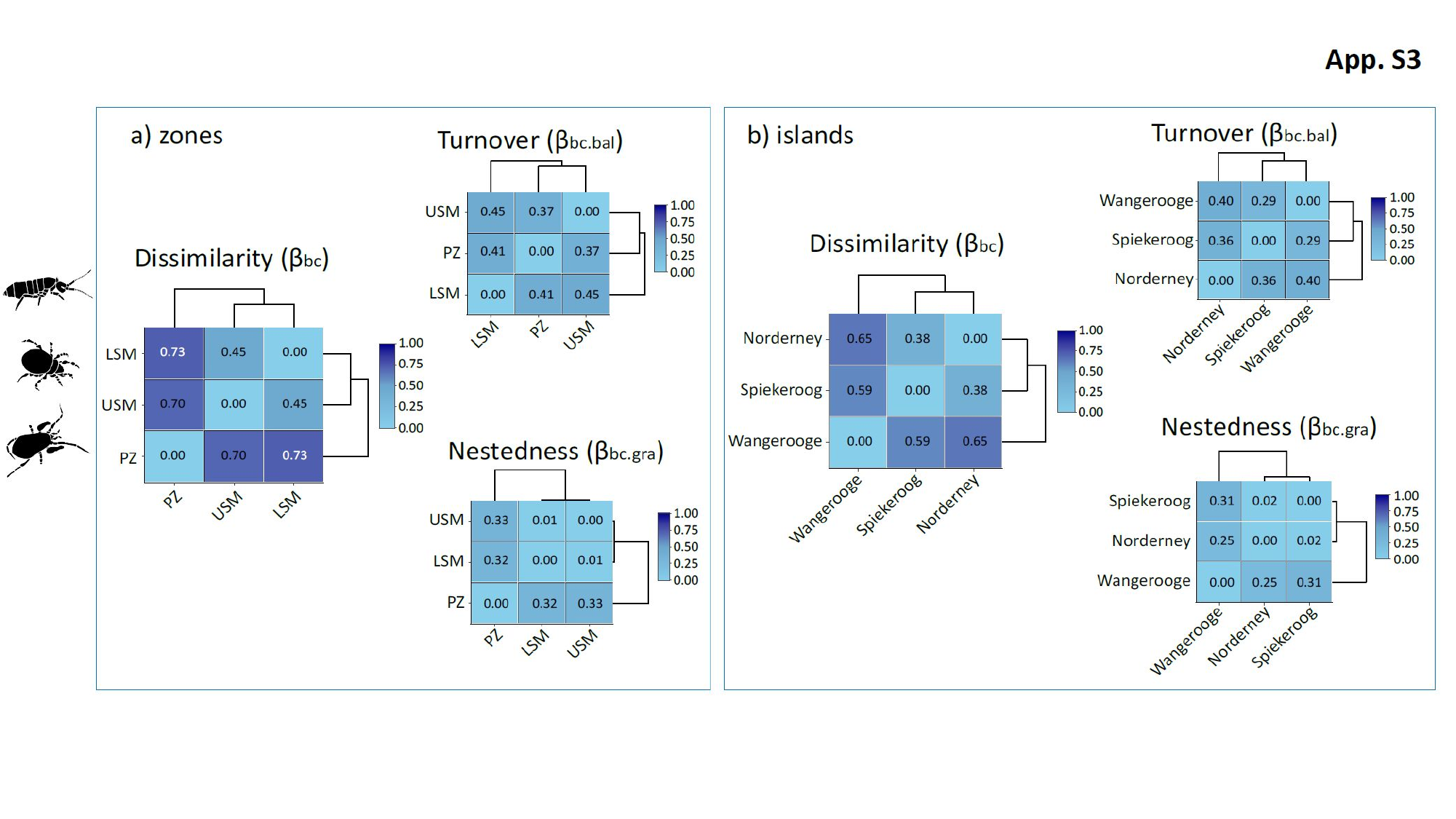

## Slide 5
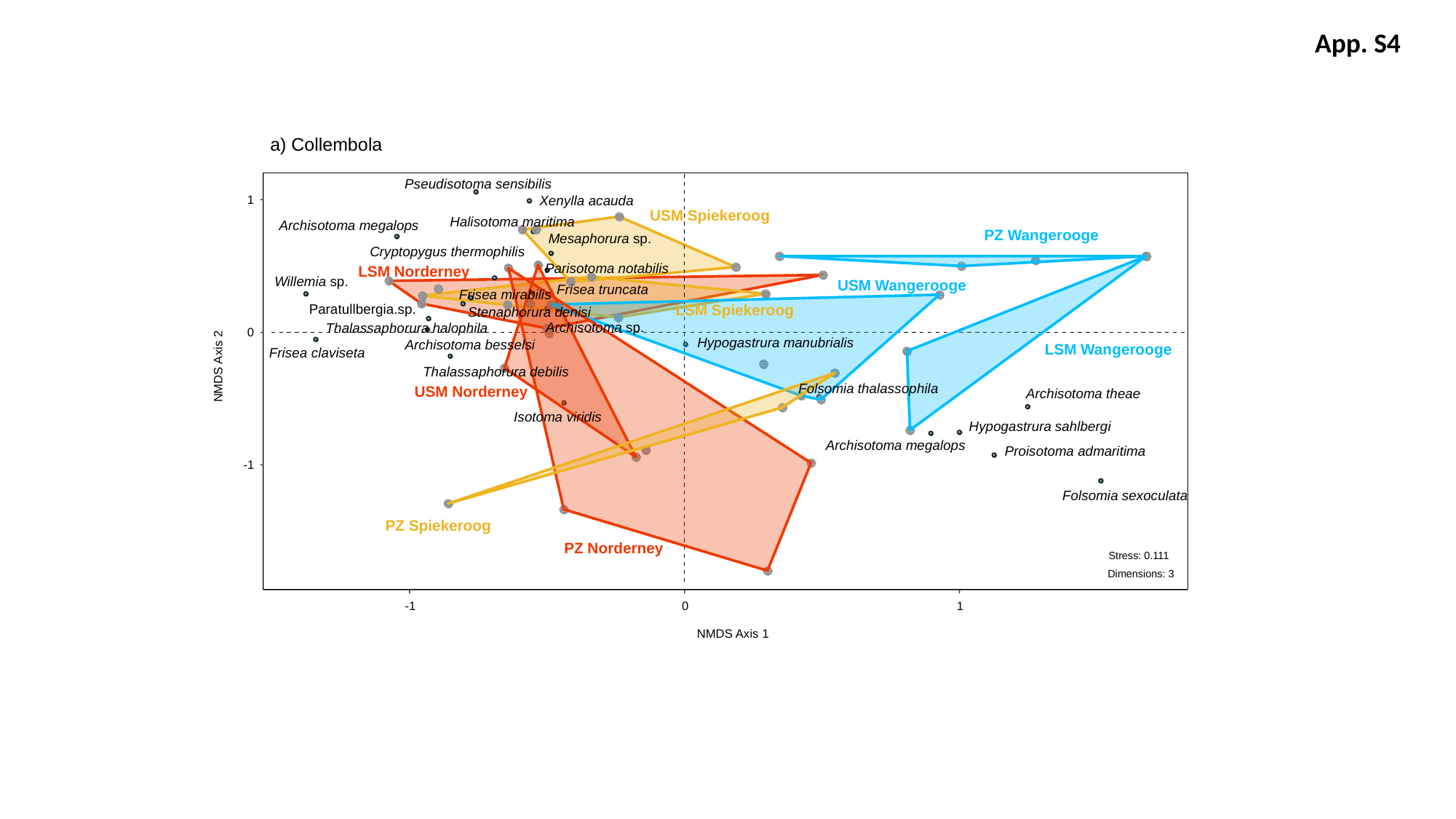

App. S4
a) Collembola
Pseudisotoma sensibilis
1
Xenylla acauda
USM Spiekeroog
Halisotoma maritima
Archisotoma megalops
PZ Wangerooge
Mesaphorura sp.
Cryptopygus thermophilis
Parisotoma notabilis
LSM Norderney
Willemia sp.
USM Wangerooge
Frisea truncata
Frisea mirabilis
LSM Spiekeroog
Paratullbergia.sp.
Stenaphorura denisi
Archisotoma sp.
Thalassaphorura halophila
0
Hypogastrura manubrialis
Archisotoma besselsi
LSM Wangerooge
Frisea claviseta
NMDS Axis 2
Thalassaphorura debilis
Folsomia thalassophila
USM Norderney
Archisotoma theae
Isotoma viridis
Hypogastrura sahlbergi
Archisotoma megalops
Proisotoma admaritima
-1
Folsomia sexoculata
 PZ Spiekeroog
PZ Norderney
Stress: 0.111
Dimensions: 3
-1
0
1
NMDS Axis 1

## Slide 6
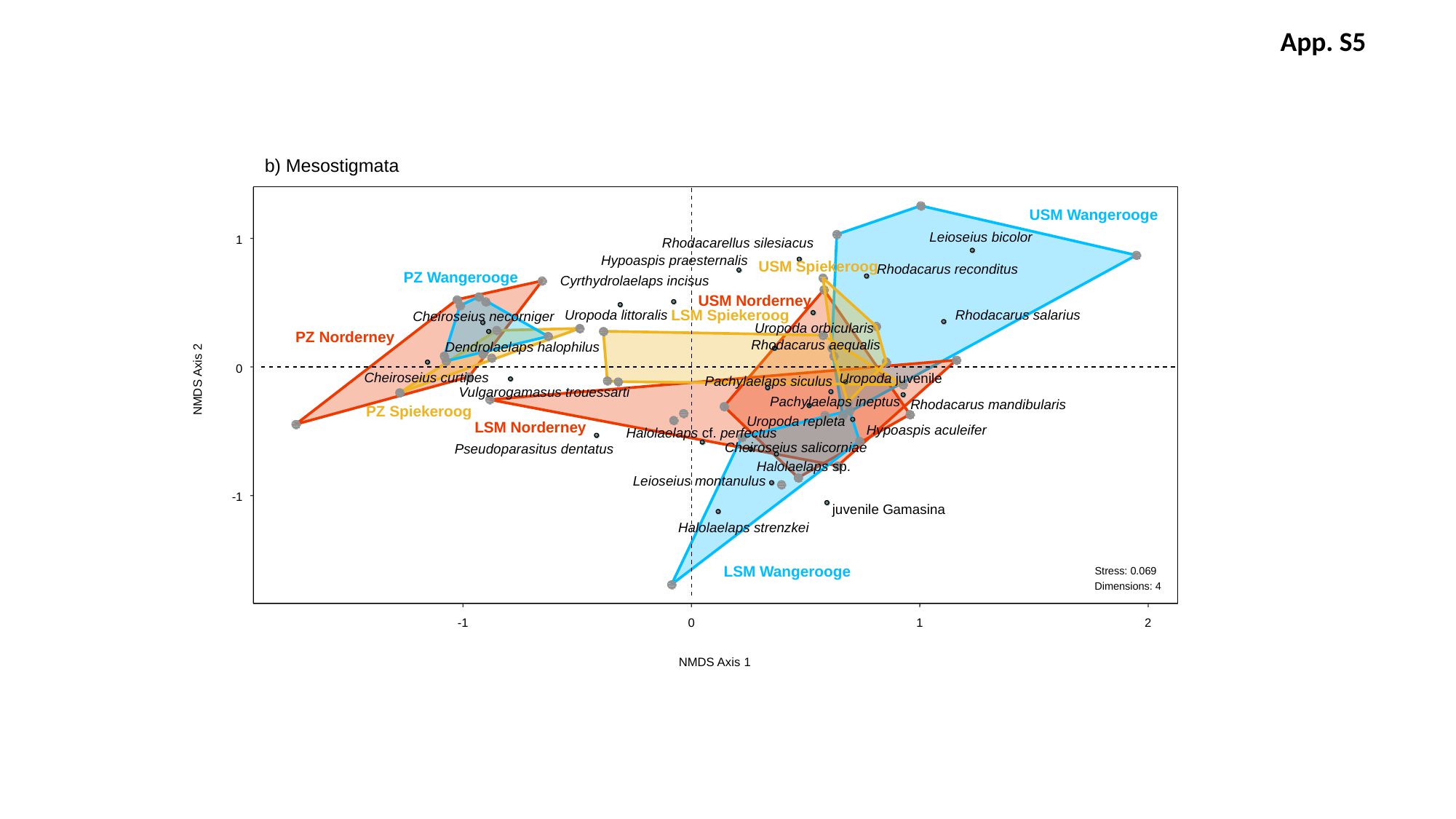

App. S5
b) Mesostigmata
USM Wangerooge
Leioseius bicolor
1
Rhodacarellus silesiacus
Hypoaspis praesternalis
USM Spiekeroog
Rhodacarus reconditus
PZ Wangerooge
Cyrthydrolaelaps incisus
USM Norderney
LSM Spiekeroog
Rhodacarus salarius
Uropoda littoralis
Cheiroseius necorniger
Uropoda orbicularis
PZ Norderney
Rhodacarus aequalis
Dendrolaelaps halophilus
0
Cheiroseius curtipes
Uropoda juvenile
NMDS Axis 2
Pachylaelaps siculus
Vulgarogamasus trouessarti
Pachylaelaps ineptus
Rhodacarus mandibularis
 PZ Spiekeroog
Uropoda repleta
LSM Norderney
Hypoaspis aculeifer
Halolaelaps cf. perfectus
Cheiroseius salicorniae
Pseudoparasitus dentatus
Halolaelaps sp.
Leioseius montanulus
-1
juvenile Gamasina
Halolaelaps strenzkei
LSM Wangerooge
Stress: 0.069
Dimensions: 4
-1
0
1
2
NMDS Axis 1

## Slide 7
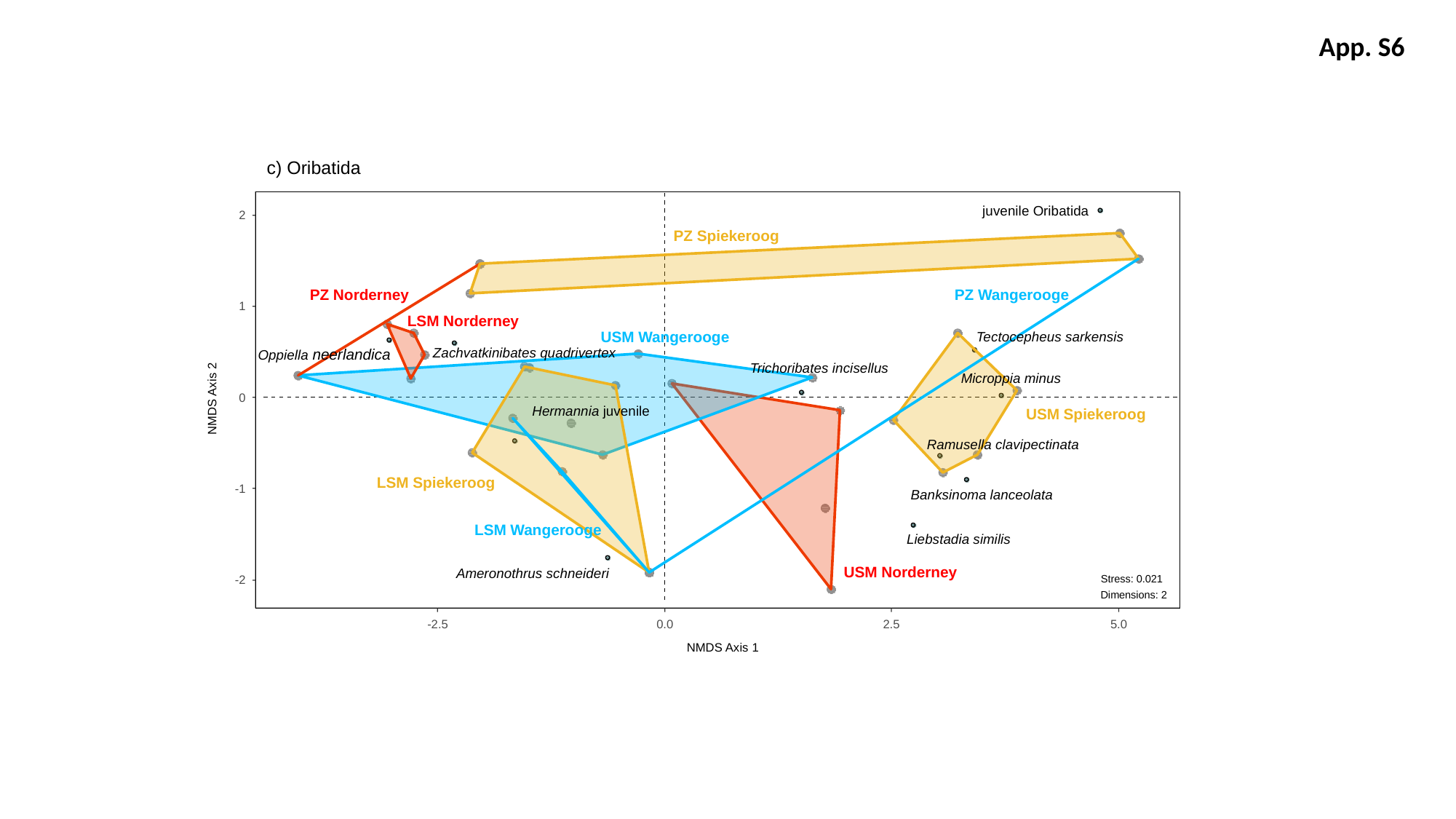

App. S6
c) Oribatida
juvenile Oribatida
2
 PZ Spiekeroog
PZ Wangerooge
PZ Norderney
1
LSM Norderney
USM Wangerooge
Tectocepheus sarkensis
Zachvatkinibates quadrivertex
Oppiella neerlandica
Trichoribates incisellus
Microppia minus
0
NMDS Axis 2
Hermannia juvenile
USM Spiekeroog
Ramusella clavipectinata
LSM Spiekeroog
-1
Banksinoma lanceolata
LSM Wangerooge
Liebstadia similis
USM Norderney
Ameronothrus schneideri
Stress: 0.021
-2
Dimensions: 2
-2.5
0.0
2.5
5.0
NMDS Axis 1

## Slide 8
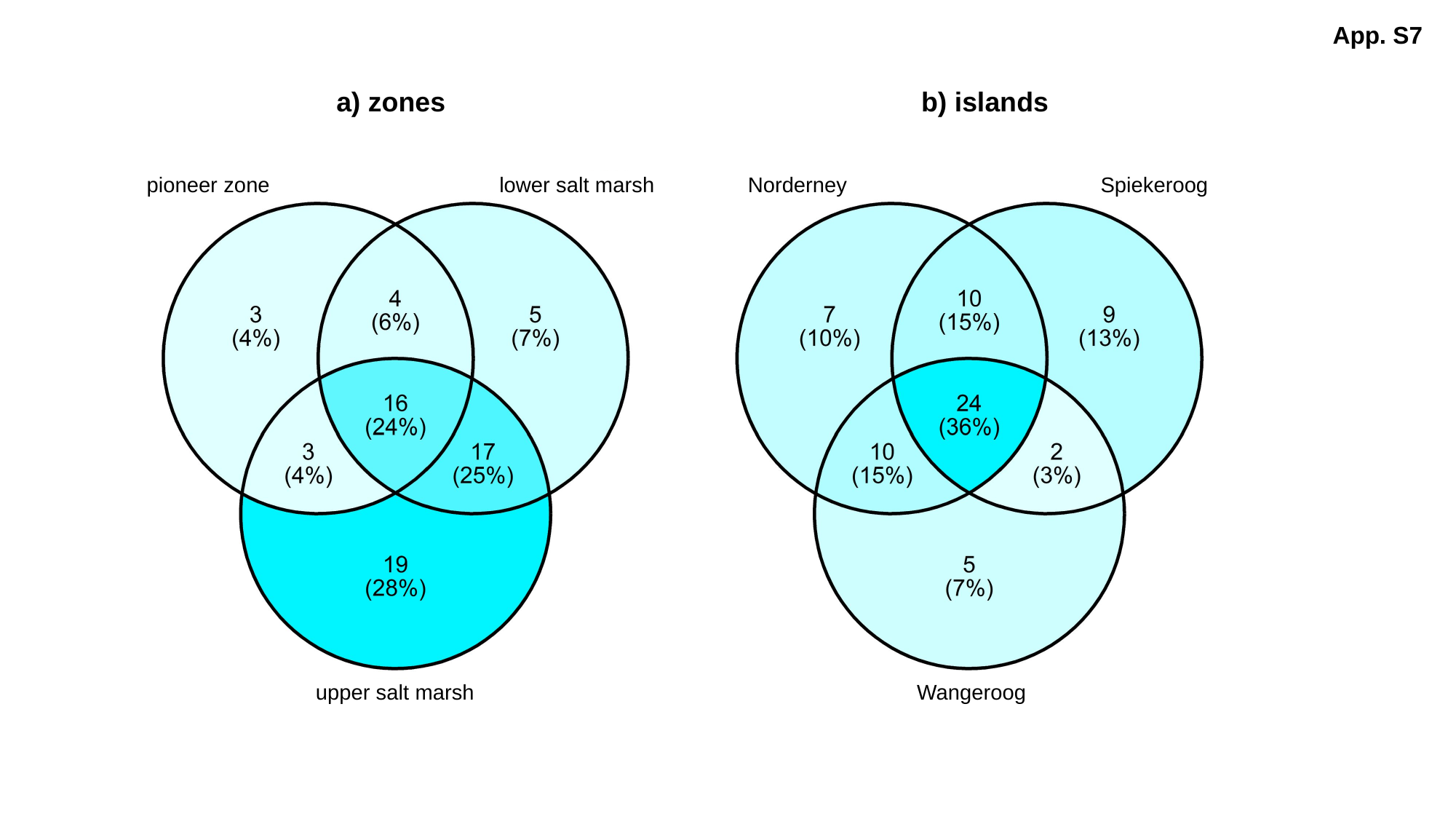

App. S7
a) zones
b) islands
Spiekeroog
PZ
Norderney
pioneer zone
lower salt marsh
Norderney
Spiekeroog
upper salt marsh
Wangeroog
